# Effects of Exendin-4 on human adipose tissue inflammation and ECM remodelling

**DOI:** 10.1038/nutd.2016.44

**Published:** 2016-12-12

**Authors:** E Pastel, S Joshi, B Knight, N Liversedge, R Ward, K Kos

**Affiliations:** 1 Diabetes and Obesity Research Group, University of Exeter Medical School, Exeter, UK; 2 NIHR Exeter Clinical Research Facility, University of Exeter Medical School, Exeter, UK; 3 RD&E NHS Foundation trust, Exeter, UK

## Abstract

**BACKGROUND/OBJECTIVES:**

Subjects with type-2 diabetes are typically obese with dysfunctional adipose tissue (AT). Glucagon-like peptide-1 (GLP-1) analogues are routinely used to improve glycaemia. Although, they also aid weight loss that improves AT function, their direct effect on AT function is unclear. To explore GLP-1 analogues' influence on human AT's cytokine and extracellular matrix (ECM) regulation, we therefore obtained and treated omental (OMAT) and subcutaneous (SCAT) AT samples with Exendin-4, an agonist of the GLP-1 receptor (GLP-1R).

**SUBJECTS/METHODS:**

OMAT and abdominal SCAT samples obtained from women during elective surgery at the Royal Devon & Exeter Hospital (UK) were treated with increasing doses of Exendin-4. Changes in RNA expression of adipokines, inflammatory cytokines, ECM components and their regulators were assessed and protein secretion analysed by ELISA. GLP-1R protein accumulation was compared in paired AT depot samples.

**RESULTS:**

Exendin-4 induced an increase in OMAT adiponectin (*P*=0.02) and decrease in elastin expression (*P*=0.03) in parallel with reduced elastin secretion (*P*=0.04). In contrast to OMAT, we did not observe an effect on SCAT. There was no change in the expression of inflammatory markers (*CD14*, *TNFA*, *MCP-1*), collagens, *TGFB1* or *CTGF*. GLP-1R accumulation was higher in SCAT.

**CONCLUSIONS:**

Independently of weight loss, which may bias findings of *in vivo* studies, GLP-1 analogues modify human OMAT physiology favourably by increasing the insulin-sensitising cytokine adiponectin. However, the reduction of elastin and no apparent effect on AT's inflammatory cytokines suggest that GLP-1 analogues may be less beneficial to AT function, especially if there is no associated weight loss.

## INTRODUCTION

Glucagon-like peptide-1 (GLP-1) is an incretin hormone produced by the intestinal L-cells from a post-translational processing of proglucagon.^1^ GLP-1 secretion is enhanced in response to nutrient ingestion and leads to a glucose-dependent increase of insulin release, which contributes to improved glucose homoeostasis. GLP-1 modulates satiety and reduces gastric emptying typically with a net effect of weight loss. GLP-1 has a short half-life owing to its rapid enzymatic degradation by dipeptidyl peptidase-4. Several analogues resistant to dipeptidyl peptidase-4 are routinely used for type-2 diabetes treatment. The concomitant weight loss also observed in non-diabetic patients led to consideration of GLP-1 analogues for obesity care.^2^

Adipose tissue (AT) is a connective tissue in which cells are embedded in a dense extracellular matrix (ECM) composed of structural proteins (collagens, elastin) and adhesion proteins (fibronectin, proteoglycans and so on), which ensure its mechanical stability, strength and elasticity.^3^ Obesity is associated with profound remodelling of AT's ECM that can lead to the establishment of fibrosis.^4^ Together with chronic inflammation, macrophage infiltration and adipocyte hypertrophy, these changes characterise AT dysfunction of obesity and insulin resistance. It is well established that inflammation as one of the key features of AT dysfunction improves with weight loss,^5^ whereas direct GLP-1 effects on human AT physiology are poorly defined and difficult to distinguish, *in vivo,* from GLP-1-induced weight loss and related improvement in glucose control as result of its incretin effect. Its receptor, GLP-1 receptor (GLP-1R), is expressed in human AT, especially by cells of the stromal vascular fraction but also by adipocytes,^6^ suggesting a role for GLP-1 on AT. Beneficial effects of GLP-1 were reported on modulation of ECM remodelling in mice myocardium showing that Exendin-4 may be able to protect from post-myocardial infarction associated interstitial fibrosis by limiting inflammation and reducing transforming growth factor β3 (TGFβ3) and collagen expression,^7^ however, the direct effects of GLP-1 on AT, especially in view of inflammation and ECM remodelling have not yet been examined.

In order to understand whether GLP-1 analogues improve AT dysfunction independent of weight loss and, especially as not all patients treated with GLP-1 analogues lose weight, the present study aims to evaluate potential effects of Exendin-4, a GLP-1 analogue, on human AT physiology in omental (OMAT) and subcutaneous AT (SCAT) explants.

## MATERIALS AND METHODS

### AT collection

OMAT and SCAT biopsies were obtained with consent from participants undergoing elective abdominal surgery at the Royal Devon & Exeter Hospital with ethics permission granted by the Royal Devon & Exeter Tissue Bank Steering Committee of the Exeter NIHR Clinical Research Facility (Exeter, UK). AT samples for explant culture were collected from seven non-diabetic overweight women with full ethical consent (seven subcutaneous abdominal with six paired OMAT biopsies). In brief, the patient characteristics of each group from this cohort were: OMAT (mean±s.d.): *n*=6, age 60.2±11.2 years, BMI 29.0±6.9 kg m^−2^, subcutaneous: *n*=7, age 60.7±10.3 years, BMI 28.4±6.5 kg m^−2^. AT samples used for protein extraction were paired OMAT and SCAT biopsies collected from eight women of which five were diagnosed with diabetes; age 51.4±6.5 years, BMI 44.6±7.8 kg m^−2^. Subjects with acute or chronic inflammatory disease, those that had undergone steroid treatment in the last 3 months or had recently taken part in a weight loss intervention/surgery were excluded.

### AT explant culture

AT was collected in Hank's Balanced Salt Solution (PAA laboratories, Pasching, Austria) supplemented with 1% 4-(2-hydroxyethyl)-1-piperazineethanesulfonic acid (Gibco, Life Technologies, Paisley, UK), 0.1% Gentamicin, 1% Amphotericin (both from Sigma-Aldrich, St Louis, MO, USA). Tissue was dissected into 5–10 mg pieces, removing blood vessels and connective tissue. Approximately 250 mg of tissue was cultured in Media 199 (Gibco, Life Technologies), supplemented with 5% fetal calf serum and 1% penicillin/streptomycin) with increasing concentrations of Exendin-4 (Isca Biochemicals, Exeter, UK), a dipeptidyl peptidase-4-resistant GLP-1R agonist sharing 53% structural homology with GLP-1.^8^ Tissue explants were incubated at 37 °C for 45 h in an atmosphere of 5% CO_2_. Media was used for analysis of protein secretion by ELISA and tissue frozen in Tri Reagent solution (Ambion, Life Technologies) for further RNA processing.

### RNA extraction

RNA was extracted using TRI Reagent according to the manufacturer's protocol. Isolated RNA was treated with DNase I (Thermo Scientific, Paisley, UK) and converted to complementary DNA using the SuperScript VILO cDNA Synthesis Kit (Invitrogen, Life Technologies) as previously described.^9^

### Gene expression analysis

Gene expression analysis was carried out using Taqman Low-Density Array cards (Life Technologies) on an ABI7900HT instrument (Applied Biosystems, Life Technologies). Relative gene expression was determined using the ΔΔC_T_ method, with expression levels normalised to the geometric mean of three housekeeping genes (*GAPDH*, *PPIA* and *UBC*). TaqMan probes used are listed in Supplementary Table 1. All samples were amplified in duplicate and data are presented as arbitrary units.

### Western blot

Tissue samples were homogenised in radioimmunoprecipitation assay buffer as previously described.^10^ Total protein (30 μg) was separated by sodium dodecyl sulfate polyacrylamide gel electrophoresis. Primary antibodies used were GLP-1 R (1:500, ab186051, Abcam, Cambridge, UK) and β-actin (1:1000, A5441, Sigma-Aldrich, Gillingham, UK). Infrared fluorescent signals were detected with relevant secondary antibodies (Li-Cor, Lincoln, NE, USA) using an Odyssey CLx and quantified with Image studio (Li-Cor).

### ELISA

Protein release in supernatant was evaluated using ELISA kits: total adiponectin, monocyte chemoattractant protein-1 (MCP-1) (both from Life Technologies) and elastin (Cloud-Clone Corp., Caltag MedSystems Ltd, Buckingham, UK). Samples were run in duplicate and absorbance measured at 450nm wavelength using a PHERAstar FS (BMG Labtech, Aylesbury, UK) microplate reader.

### Statistical analysis

All data are presented as the mean±s.e.m. unless otherwise stated. Assessment of the dose response of Exendin-4 was analysed using Friedman's one-way analysis of variance followed by a Dunn's *post hoc* test to correct for multiple testing. For western blot analysis, statistical significance was assessed using a Wilcoxon test. Statistics were performed using GraphPad Prism 5.04 software (La Jolla, CA, USA), *P*-values<0.05 were considered significant.

## RESULTS

In the following sections, we studied the effect of GLP-1R agonists on human OMAT and SCAT by studying the dose response to Exendin-4 treatment *ex vivo*.

### Adipokines

**Figure 1:**
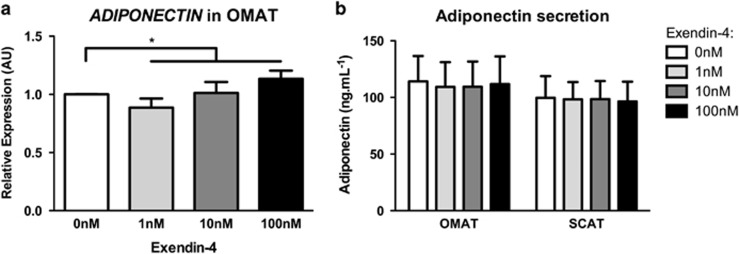
Exendin-4 stimulated adiponectin expression in OMAT explants. (**a**) Omental (OMAT) AT's adiponectin mRNA expression as result of increasing doses of Exendin-4 (0–100 nm). (**b**). Adiponectin concentrations in supernatant obtained from culture of OMAT and subcutaneous adipose tissue (SCAT) explants with Exendin-4. *P<0.05, OMAT, *n*=6; SCAT, *n*=7.

Adiponectin (*ADIPOQ*) exerts cardioprotective, insulin-sensitising and anti-inflammatory effects.^11^ AT explant treatment with Exendin-4 increased *ADIPOQ* expression in OMAT (Figure 1a and Table 1) with no effect in SCAT (Table 1). To confirm this observation, we measured adiponectin levels in supernatant obtained from explant culture. We did not observe any modification of adiponectin secretion in OMAT or SCAT (Figure 1b). Leptin is involved in the regulation of satiety, energy expenditure, body weight and insulin sensitivity.^11^ *LEP* expression was not significantly altered by Exendin-4 in either OMAT or SCAT explants (Table 1).

### Inflammatory cytokines

Monocyte chemoattractant protein-1 (MCP-1) and tumour necrosis factor alpha (TNFα) are produced by both adipocytes and AT resident macrophages and CD14 (cluster of differentiation 14) is a monocyte/macrophage marker expression of which reflects macrophage infiltration in AT.^12^ MCP-1 (also called CCL2; CC-chemokine ligand 2) is involved in immune cell recruitment into AT. Its expression was not significantly affected by increasing doses of Exendin-4 in either OMAT, or SCAT. We confirmed this observation by analysis of its protein secretion in the explant media and found no change (data not shown). Similarly, we did not find any changes in *TNFA* or *CD14* expression in response to Exendin-4 (see Table 1). We also did not find any modification in the expression of inflammatory markers after treatment with Insulin with or without Exendin-4 (data not shown).

### Fibrosis: ECM and its regulators

**Figure 2:**
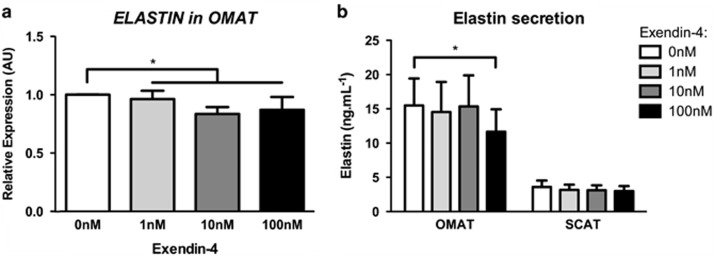
Exendin-4 treatment decreased expression and secretion of elastin in OMAT. (**a**) Accumulation of elastin mRNA in OMAT after treatment with 0, 1, 10, 100 nM of Exendin-4 (OMAT, *n*=6; SCAT, *n*=7). (**b**) Elastin release into media of OMAT and SCAT in response to Exendin-4 treatments and significant for comparison of 0 nm vs 100 nM (OMAT, *n*=9; SCAT, *n*=7). *P<0.05.

In order to assess Exendin-4's impact on AT's ECM composition, we analysed expression of ECM main proteins (collagens, fibronectin and elastin), enzymes involved in their cross-linking (LOX, lysosyl oxidase and LOXL2, lysyl oxidase-like 2) and degradation (MMPs, metalloproteinases) as well as regulators of ECM remodelling (TGFβ1, transforming growth factor beta 1 and CTGF, connective tissue growth factor). We analysed expression of the three main collagen types found in AT: *COL1A1* (collagen 1 alpha subunit 1), *COL3A1* (collagen 3 alpha subunit 1) and the pericellular *COL4A1* (collagen 4 alpha subunit 1).^9^ We did not find any significant changes in the expression of these collagen subunits in OMAT or SCAT (Table 1). Similarly, fibronectin (*FN1*) did not appear to be affected by Exendin-4 treatment. In contrast to the collagens, elastin fibres provide stretchiness to AT.^3^ We found that Exendin-4 treatment significantly decreased elastin (*ELN*) expression within OMAT explants, however, this effect was not seen within SCAT. We therefore assessed elastin synthesis by measuring its release in culture media obtained from AT explants treated with Exendin-4. Exposure of AT explants to high dose of Exendin-4 significantly decreased elastin synthesis in OMAT explants but not SCAT (Figure 2b).

LOX and LOXL2 are two enzymes involved in collagen and elastin cross-linking.^13^ We found that neither *LOX* nor *LOXL2* responded to Exendin-4. Similarly, we observed no change on expression of two key enzymes involved in ECM break down: *MMP9* and *MMP14*. Moreover in response to Exendin-4 treatment, we monitored no change in the two pro-fibrotic messengers: *TGFB1* and *CTGF* (Table 1).

### Other proteins relevant to AT function

We assessed the expression of *PPARG* (peroxisome proliferator-activated receptor gamma), one of the main regulator of adipogenesis and did not observe any modification, nor did we find a change in the hypoxia marker *HIF1A* (hypoxia inducible factor 1 alpha) with hypoxia being one of the mediators of fibrosis and inflammation.^14^ Also, there were no changes in the lipogenesis regulator *LPL* (lipoprotein lipase) or the endothelial factor *CD31* (cluster of differentiation 31)/*PECAM1* (platelet/endothelial cell adhesion molecule 1, see Table 1).

### GLP-1R expression in OMAT and SCAT

**Figure 3:**
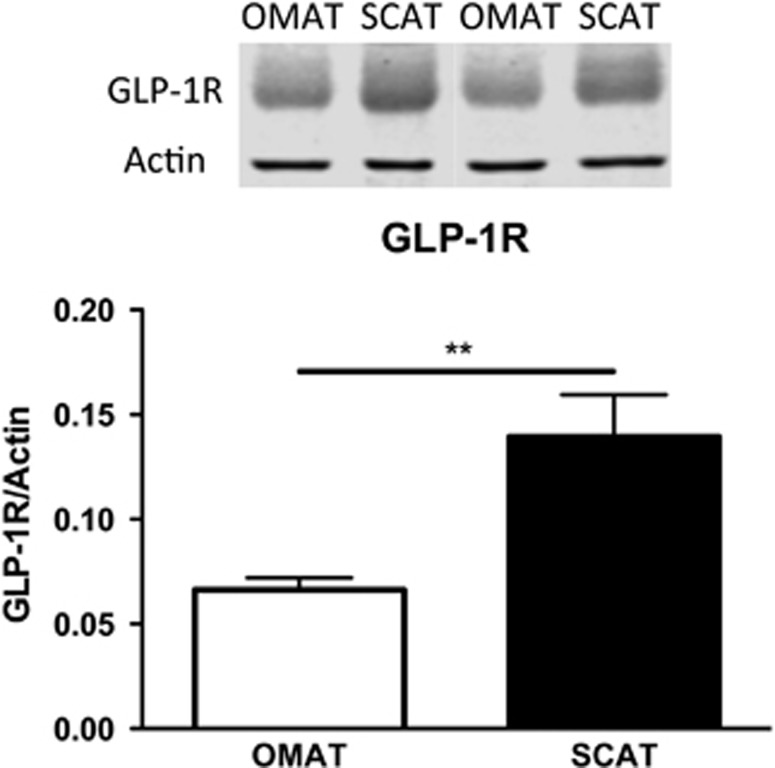
GLP-1R expression is stronger in SCAT than in OMAT. Accumulation of GLP-1R protein in OMAT and SCAT with actin as a loading control. **P<0.01, OMAT and SCAT *n*=8.

Owing to the marked differences in the depot response to Exendin-4 of both adiponectin and elastin, we further examined whether this could be due to a difference in receptor expression. Using a cohort of paired OMAT and SCAT samples (*n*=8), we observed, by western blot, a stronger accumulation of GLP-1R in SCAT (Figure 3).

## DISCUSSION

We examined direct effects of GLP-1 analogues on abdominal AT depots, which are prone to expand with increasing obesity and susceptible to AT dysfunction.^15^ Our study shows that acute treatments based on GLP-1 analogues may have an advantage by increasing adiponectin expression and that they reduce elastin secretion by which they take part in ECM remodelling. Studying whole AT, with its stromal vascular fraction, enables to account for the cross-talk between its different cell populations. Preadipocytes and adipocytes both express GLP-1R^6^ and are involved in ECM protein production. This model allowed us for the first time to study effects of GLP-1 on the ECM without the influence of weight loss or glycaemic improvement which are recognized effects of GLP-1 analogue treatments.

### GLP-1 and adiponectin

In response to Exendin-4, we found a dose-dependent increase of adiponectin expression in OMAT explants. Adiponectin is, mainly secreted by adipocytes, with plasma concentrations inversely correlated with insulin resistance, type-2 diabetes and obesity.^11^ Adiponectin has insulin-sensitising and anti-inflammatory properties and an ability to enhance pancreatic β-cells regeneration.^16^ Though adiponectin is mainly secreted by SCAT, Drolet *et al.*^17^ found that the OMAT release of adiponectin was reduced with increasing BMI, total body fat mass and visceral AT area, suggesting that OMAT could be responsible for the hypoadiponectinemia associated with obesity. Treatments of patients with GLP-1R agonists promote weight loss and increase circulating adiponectin levels.^18^ However, little is so far known about GLP-1 treatment effects on human AT *in vivo* controlled for weight change, apart from a recent study in which Exenatide was started in type-2 diabetic subjects prior to bariatric surgery. This study has shown similar to us, a selectively increase of adiponectin expression in visceral not SCAT.^19^ In line with these findings, our results go further and show that Exendin-4 treatment changes adiponectin expression in OMAT has yet little effect on adiponectin secretion. Thus, the increased circulating adiponectin secretion as previously reported with GLP-1 treatment is mainly a feature of weight loss.^20^ An accumulative effect, stronger with chronic treatment, cannot be excluded. Murine studies postulate activation of a PKA-dependent pathway^21^ and M2 macrophage polarisation^22^ as potential pathways by which GLP-1 may stimulate adiponectin expression.

### GLP-1 and ECM

Weight loss is accompanied by profound ECM remodelling. *In vitro* studies allow assessment of GLP-1 effects independent of weight loss. We showed a decrease of elastin expression in OMAT in response to Exendin-4, whereas fibre forming type I, II and IV collagens did not appear to be affected. Elastin confers resilience, elasticity and deformability to many connective tissues including AT.^3^ Elastin was reported to be less abundant in AT from non-diabetic obese than lean subjects when assessed by immunohistology.^23^ Furthermore, mice with haplo-insufficiency of the *elastin* gene in a *ApoE*^−^/_−_ background had impaired glucose metabolism and demonstrated adipocyte hypertrophy,^24^ supporting that elastin production is impaired in obesity and that it may have a role in glucose metabolism. The significance of the Exendin-4 induced decrease of elastin expression and secretion on AT function needs yet to be further clarified. Mechanical properties of ECM result from a balance between relative abundance of elastin and collagen, their efficient cross-linking by LOX and LOXL2 and their degradation, which is influenced by metalloproteinases and their inhibitors. Apart from the change in elastin, we did not observe any modification of *LOX*, *LOXL2* or *MMPs* expression in response to Exendin-4 treatment, and cannot conclude an effect of GLP-1 to histological changes or mechanical properties of AT's ECM, as technically not feasible. Similarly, the expression of *TGFB1* and its downstream target *CTGF*, two key pro-fibrotic factors were not affected by Exendin-4, suggesting that GLP-1 does not induce a pro-fibrotic phenotype, though a change in the mechanical property by changing the ratio of collagen/elastin^3^ cannot be excluded.

### GLP-1 and inflammation

Chronic low-grade inflammation and macrophage infiltration in AT have been reported to be closely related to the development of obesity and insulin resistance.^25^ We analysed the expression of inflammation markers in response to Exendin-4 treatment and found no change in either abdominal depot. Most importantly, we did not find changes in *MCP-1* expression or secretion, whereas some studies suggest that 12 weeks treatment of obese patients with type-2 diabetes with the GLP-1R agonists, Exenatide decreases circulating cytokines including MCP-1 plasma concentrations independent of weight change, however, with a significant improvement in glycaemia.^26^ Other reports show that GLP-1 analogues inhibit expression of inflammatory cytokines in 3T3-L1 adipocytes.^21^ Nevertheless, anti-inflammatory properties of GLP-1 analogues have not been confirmed as direct effect and specific to human AT and several groups have seen no modification in MCP-1 or other cytokines' plasma concentrations (which are likely to be attributable to AT inflammation) in non-obese subjects with type-2 diabetes despite weight loss,^18^ nor a change in AT expression of *TNFα* or *MCP-1* in non-diabetic rats treated with Exenatide.^27^ An obvious direct effect of GLP-1 on AT inflammation independent of weight loss effects thus remains questionable. Clinical studies controlling for the improvement in glycaemia and the effect of weight loss, for example, by comparing patients with dietary induced weight loss of similar magnitude to the GLP-1 induced weight loss are needed.

### Differential AT depot response

We found only OMAT responsive to Exendin-4 treatment. OMAT and abdominal SCAT exhibit different patterns of gene expression,^28^ OMAT has higher lipolytic activity^15^ and has as part of visceral AT a clear link with the development of metabolic syndrome characteristics (including glucose intolerance, hyperinsulinemia and hypertriglyceridemia) and cardiovascular disease.^15^ We postulated that the difference between OMAT and SCAT response could have been due to a differential expression of GLP-1R. We found more GPL1-R protein in SCAT, demonstrating that the different response to Exendin-4 is unlikely explained by the amount of GLP-1R. Higher expression of GLP-1R was found in OMAT of obese insulin-resistant subjects when compared with subjects with low insulin resistance,^6^ however, we were not able to reproduce these findings in morbidly obese subjects of whom most had diabetes. GLP-1R resistance in combination with insulin resistance cannot be excluded.

We acknowledge some study limitations. We may have missed peaks in mRNA expression occurring prior to 45 h of tissue culture and/or later mRNA changes. The duration was in part chosen to enable the study of cytokine and protein accumulation in the media. Similar to other *in vitro* studies,^6, 7^ we used 1–100 nM of Exendin-4 in our experiments. Even if previous pharmacokinetic studies showed that Exendin-4 treatment with current dosing results in circulating levels between 2.5 and 5 nM,^29^ higher doses of GLP-1 analogues are now under consideration for weight management.^2^ AT samples were used from non-diabetic overweight/obese subjects who are typically insulin resistant, whereas GLP-1 treatment is primarily for subjects with type-2 diabetes. We have expected an improvement in AT inflammation as reported in rodents and humans (circulatory/plasma inflammatory markers),^26, 30^ which we were unable to confirm and did not expect the need to stimulate inflammation in this *in vitro* explant model. Further studies are necessary, which include a model to take account of macrophage infiltration and polarisation within explant culture. We used normoglycaemic conditions as will be achieved by effective treatment of diabetes with GLP-1 analogues or when used in non-diabetic subjects for weight management. Furthermore, we were only able to study acute effects and can only hypothesise that they may become more pronounced with prolonged treatment.

In summary, Exendin-4 can directly modify expression of adiponectin and elastin in explant culture of human AT. Interestingly, OMAT, expansion of which is linked to the development of insulin resistance and the metabolic syndrome, seems to be more sensitive to Exendin-4, despite higher GLP-1 receptor expression in subcutaneous tissue. These results suggest that treatment of patients with type-2 diabetes and/or obesity with GLP-1 analogues could directly influence AT physiology, its properties and functions. However, apart from the positive effect on increased adiponectin expression/secretion we cannot verify an anti-inflammatory effect of GLP-1 on AT independent of weight loss.

## Figures and Tables

**Table 1:** Gene expression changes in adipose tissue explants following treatment with Exendin-4

*Exendin-4*	*OMAT*				*SCAT*			
	*1 nm*	*10 nm*	*100 nm*	P-*value*	*1 nm*	*10 nm*	*100 nm*	P-*value*
*Adipokines*								
*ADIPOQ*	0.89±0.08	1.01±0.09	1.13±0.07	**0.02**	1.01±0.06	1.05±0.04	1.05±0.05	0.69
*LEP*	1.00±0.12	1.09±0.10	1.00±0.17	0.84	0.89±0.09	0.93±0.05	0.99±0.08	0.32
								
*Inflammation*								
*CD14*	0.91±0.09	1.08±0.11	0.97±0.09	0.32	1.03±0.07	1.00±0.06	0.94±0.09	0.42
*MCP-1*	0.97±0.06	0.97±0.07	0.93±0.08	1.00	0.86±0.10	0.86±0.09	0.93±0.09	0.48
*TNFA*	0.96±0.07	1.11±0.15	0.98±0.12	0.77	0.96±0.06	0.91±0.06	1.03±0.04	0.48
								
*ECM and its regulators*								
*COL1A1*	1.00±0.10	0.94±0.05	0.91±0.09	0.54	0.97±0.09	1.02±0.06	0.98±0.08	0.86
*COL3A1*	0.92±0.07	0.95±0.07	0.91±0.08	0.43	0.99±0.08	1.06±0.07	1.02±0.05	0.42
*COL4A1*	0.95±0.08	0.99±0.10	1.01±0.10	0.87	1.02±0.08	1.07±0.04	1.03±0.06	0.61
*CTGF*	1.05±0.15	0.92±0.13	1.00±0.17	0.16	0.93±0.05	0.92±0.07	1.01±0.06	0.54
*ELN*	0.96±0.07	0.84±0.06	0.87±0.11	**0.03**	0.95±0.07	0.92±0.06	0.98±0.11	0.86
*FN1*	1.04±0.09	0.95±0.10	1.06±0.09	0.32	0.94±0.11	0.87±0.06	0.86±0.06	0.18
*LOX*	0.95±0.08	0.93±0.07	0.84±0.04	0.16	0.96±0.05	0.97±0.05	0.95±0.05	0.86
*LOXL2*	0.91±0.09	0.90±0.11	0.97±0.07	0.94	0.98±0.04	1.03±0.02	1.02±0.05	0.61
*MMP9*	1.09±0.13	0.92±0.15	0.77±0.09	0.09	1.01±0.19	0.91±0.07	0.98±0.08	0.82
*MMP14*	0.98±0.09	0.97±0.05	0.91±0.06	0.67	0.98±0.06	0.94±0.06	0.97±0.08	0.86
*TGFB1*	0.95±0.09	0.93±0.07	0.92±0.04	0.57	0.95±0.05	0.95±0.05	0.96±0.08	0.86
								
*Others*								
*CD31*	0.85±0.08	0.88±0.10	0.94±0.07	0.77	1.01±0.08	0.99±0.04	0.99±0.05	0.93
*HIF1A*	0.93±0.07	0.92±0.06	0.90±0.06	0.26	0.98±0.03	0.90±0.06	0.96±0.05	0.12
*LPL*	0.93±0.06	1.05±0.06	1.04±0.08	0.38	1.04±0.04	0.98±0.02	1.06±0.04	0.09
*PPARG*	0.91±0.06	1.04±0.11	1.05±0.08	0.51	1.02±0.07	1.03±0.05	1.06±0.07	0.82

Abbreviations: ECM, extracellular matrix; OMAT, omental; SCAT, subcutaneous adipose tissue. Expression of genes in human OMAT (*n*=6) and SCAT (*n*=7) explants treated 45 h with increasing doses of Exendin-4. Data were normalised to the control condition and expressed as the mean±s.e.m. Significant *P*-values are in bold.
