# Supplementary material for: Effects of Exendin-4 on human adipose tissue inflammation and ECM remodelling
Source: Nutr Diabetes. 2016 Dec 12;6(12):e235–. doi: 10.1038/nutd.2016.44 (PMC5223133; doi:10.1038/nutd.2016.44)
Supplement: Supplementary Information [file nutd201644x1.docx]

**Supplemental table 1.** List of TaqMan primer/probe assays.

| **Gene name** | **Gene symbol** | **Assay ID** |
| --- | --- | --- |
| **Adipokines** | | |
| Adiponectin | *ADIPOQ* | Hs00605917_m1 |
| Leptin | *LEP* | Hs00174877_m1 |
| **Inflammations** | | |
| CC-chemokine ligand 2 | *CCL2/MCP-1* | Hs00234140_m1 |
| Cluster of differentiation 14 | *CD14* | Hs02621496-s1 |
| Tumour necrosis factor alpha | *TNFA* | Hs99999043_m1 |
| **ECM and its regulators** | | |
| Collagen 1 alpha subunit 1 | *COL1A1* | Hs00164004_m1 |
| Collagen 3 alpha subunit 1 | *COL3A1* | Hs00943809_m1 |
| Collagen 4 alpha subunit 1 | *COL4A1* | Hs00266237_m1 |
| Connective tissue growth factor | *CTGF* | Hs01026927_g1 |
| Elastin | *ELN* | Hs00355783_m1 |
| Fibronectin | *FN1* | Hs00365052_m1 |
| Lysyl oxidase | *LOX* | Hs00942480_m1 |
| Lysyl oxidase-like 2 | *LOXL2* | Hs00158757_m1 |
| Matrix metalloproteinase 9 | *MMP9* | Hs00234579_m1 |
| Matrix metalloproteinase 14 | *MMP14* | Hs00237119_m1 |
| Transforming growth factor beta 1 | *TGFB1* | Hs00998133_m1 |
| **Others** | | |
| Cluster of differentiation 31 | *CD31/PECAM1* | Hs00169777_m1 |
| Hypoxia inducible factor 1 alpha | *HIF1A* | Hs00153153_m1 |
| Lipoprotein lipase | *LPL* | Hs00173425_m1 |
| Peroxisome proliferator-activated receptor gamma | *PPARG* | Hs01115513_m1 |
| **Housekeeping genes** | | |
| Glyceraldehyde-3-phosphate dehydrogenase | *GAPDH* | Hs99999905_m1 |
| Peptidylprolyl isomerase A (cyclophilin A) | *PPIA* | Hs99999904_m1 |
| Ubiquitin C | *UBC* | Hs00824723_m1 |
